# Genomic Features of *Salmonella enterica* Subspecies *houtenae* Serotype 45:g,z51:- Isolated from Multiple Abdominal Abscesses of an African Fat-Tailed Gecko, United States, 2020

**DOI:** 10.3390/antibiotics10111322

**Published:** 2021-10-29

**Authors:** Ji-Yeon Hyeon, Zeinab H. Helal, Robert Polkowski, Kristin Vyhnal, Neha Mishra, Junwon Kim, Guillermo R. Risatti, Dong-Hun Lee

**Affiliations:** 1 Department of Pathobiology and Veterinary Science, College of Agriculture, Health and Natural Resources, University of Connecticut, Storrs, CT 06269, USA; jiyeon.hyeon@uconn.edu (J.-Y.H.); zeinab.helal@uconn.edu (Z.H.H.); Kristin.Vyhnal@uconn.edu (K.V.); neha.mishra@uconn.edu (N.M.); junwon.kim@uconn.edu (J.K.); guillermo.risatti@uconn.edu (G.R.R.); 2 Connecticut Veterinary Medical Diagnostic Laboratory, Department of Pathobiology and Veterinary Science, College of Agriculture, Health and Natural Resources, University of Connecticut, Storrs, CT 06269, USA; robert.polkowski@uconn.edu

**Keywords:** *Salmonella enterica* subspecies *houtenae*, reptile, African fat-tailed gecko, complete genome sequence, whole genome sequencing

## Abstract

*Salmonella enterica* subsp. *houtenae* (*S. houtenae*) is a common subspecies in reptiles and has been implicated as a source of serious and life-threatening diseases in humans. Although occurrence and significance of *S. houtenae* infections have been extensively studied, the genetic features of *S. houtenae* have remained unknown due to a lack of available high-quality genome sequences. We obtained the complete genome sequence of *S. houtenae* 45:g,z51:- strain 20-369 isolated from multiple abdominal abscesses of an African fat-tailed gecko (*Hemitheconyx caudicinctus*) using Nanopore and Illumina sequencing technologies and generated the 4.65Mbp complete genome sequence of the *S. houtenae* str. 20-369. We annotated and analyzed the genome sequence with the aim to gain a deeper understanding of the genome characteristics associated with its pathogenicity. Overall, this study found several interesting genomic features such as pseudogene formation, virulence gene profile, and novel genomic islands. This study provides basis for an understanding possible genetic mechanism underlying pathogenicity of *S. houtenae* 45:g,z51:- as well as a high-quality genome reference for future comparison studies.

## 1. Introduction

*Salmonella* is a genus of Gram-negative, non-sporulated and facultative anaerobe bacillus with flagella and mobility, composed of 2579 different serotypes [1]. Based on the 16S rRNA sequence and biochemical analysis, *Salmonella* is divided into two species: *Salmonella enterica* (*S. enterica*) and *Salmonella bongori* (*S. bongori*) [1]. *S. enterica* is then divided into six different subspecies, each designated with Roman numeral: *enterica* (I), *salamae* (II), *arizonae* (IIIa), *diarizonae* (IIIb), *houtenae* (IV) and *indica* (VI) [2]. Most *Salmonella* diseases are linked to a wide variety of serotypes of *S. enterica subsp*. *enterica* (I), being its main route of dissemination contaminated food and water [3]. However, the participation of *Salmonella* subsp. II-VI in cases of atypical diseases in humans, has recently been described [4,5].

*S. enterica* subsp. *houtenae* (*S. houtenae*) was originally isolated from a cockatiel (*Nymphicus hollandicus*) in 1978, and 73 serotypes of *S. houtenae* have been described since [6,7]. *S. houtenae* inhabits the intestinal tract of reptiles and has been found to be prevalent in terrestrial and aquatic turtles, snakes, land Iguana, Australian sleepy lizards and captive zoo reptiles [1,4,8]. *S. houtenae* is the most prevalent subspecies identified from 31 cases of *Salmonella* infection in human with known exposure to reptiles in Germany between 2006 and 2008 [4]. *S. houtenae* has been reported as an opportunistic pathogen in humans, and some serovar of *S. houtenae* have been implicated as a source of serious and life-threatening diseases in humans, such as sepsis, meningitis, brain abscesses endocarditis and urinary tract infections that primarily affect children aged less than one year and immunocompromised adults [3,4,5,7,9,10,11,12,13]. However, the pathogenic potential of *S. houtenae* has been underestimated [14], and there is only very limited genomic information about *S. houtenae*. As of 8 March 2021, only six complete genome sequences of *S. houtenae* have been reported in NCBI GenBank database; *S. houtenae* serotypes 16:z4,z32:-, 44:z4,z23:-, and 43:z4,z23:-, and to our best knowledge, there is only two published reports of genome analysis of *S. houtenae* but no reports of complete genome analysis [8,14].

According to the report on laboratory-confirmed *Salmonella* infections during 2003–2013 by Centers for Disease Control and Prevention (CDC) [15], *S. houtenae* serotypes 50:z4,z23:-, 50:g,z51:-, 48:g,z51:-, 44:z4,z23:-, and 45:g,z51:- are the most prevalent in *S. houtenae* infection. In this study, we produced the first complete genome sequence of *S. houtenae* 45:g,z51:- strain 20-369 isolated from multiple abdominal abscesses of an African fat-tailed gecko in Connecticut, United States. We analyzed the genomic features of the isolate including presence of antibiotic resistance genes and chromosome mutations, pseudogenes, plasmids, virulence gene profiles, and single-nucleotide polymorphism (SNP) to establish phylogenetic relationships with other *Salmonella* spp.

## 2. Methods

### 2.1. Bacterial Isolation and Identification

*S. houtenae* str. 20-369 was isolated from multiple abdominal abscesses of a 3-year-old female African fat-tailed gecko (*Hemitheconyx caudicinctus*) at the Connecticut Veterinary Medical Diagnostic Laboratory (CVMDL), Department of Pathobiology and Veterinary Science, University of Connecticut in 2020. The African fat-tailed gecko had severe subacute pyogranulomatous and necrotizing oophoritis and minimal to moderate multifocal granulomatous peritonitis with intralesional bacterial colonies, and histopathology revealed infection originating in the ovary. For isolation of *Salmonella* spp. from the abdominal abscesses, the clinical samples were plated on brilliant green novobiocin (BGN), Xylose Lysine Tergitol-4 (XLT-4) and blood agar plates, then the plates were incubated T Dickinson, Franklin Lakes, NJ, USA) and via 16S rRNA PCR amplification and a sequence alignment of the amplicon using the Basic Local Alignment Search Tool (BLAST).

### 2.2. Antibiotic Susceptibility Test of S. houtenae Isolate

The Kirby-Bauer Test disc-diffusion method was performed to determine antibiotic susceptibility of the isolate as recommended by Clinical and Laboratory Standards Institute for a consensus interpretive criterion [16]. The antibiotics tested in this study are listed in Table 1.

### 2.3. Whole Genome Sequencing

Two separate genomic DNA libraries were prepared according to the requirements of the Illumina and Oxford Nanopore systems. A combination of long-read Nanopore MinION and short-read Illumina MiSeq was used to generate the complete genome sequence of *S. houtenae* str. 20-369. For short-read sequencing, genomic DNA was extracted from pure cultures of the isolate using the DNeasy Blood and Tissue kit (Qiagen, Valencia, CA, USA) according to the manufacturer’s instructions. DNA samples (0.2 ng/μL) were used for the library preparation using the Illumina Nextera XT DNA Library Prep Kit (Illumina, San Diego, CA, USA). The libraries were diluted to a 2 nM concentration using the Qubit BR dsDNA HS assay kit and combined in equal volumes to form the pooled library. The library pool (600 µL of the 10 pM libraries) was loaded into the MiSeq reagent v2 250 cycle cartridge (Illumina, San Diego, CA, USA). The paired FASTQ files were base called from the Illumina raw sequence read data. The raw sequence reads were trimmed using Trimmomatic v0.36.6 to trim sequencing adapters, reads with a quality score <30 over a sliding window size of 4 bp, and reads with a sequence length <50 bp [17]. After trimming the adaptors and filtering low-quality reads, the clean sequence data were used for further bioinformatics analyses.

For Nanopore sequencing, genomic DNA was extracted from pure cultures of *S. houtenae* using the Wizard^®^ HMW DNA Extraction Kit (Promega, Madison, WI, USA) according to the manufacturer’s instructions. A MinION sequencing library was prepared using the Rapid Barcoding Sequencing Kit (SQK-RBK004; Oxford Nanopore, Oxford, UK). The library was sequenced with an R9.4.1 MinION flow cell (FlO-MIN106, Oxford Nanopore, Oxford, UK) for 48 h using MinKNOW v2.0 with the default settings. FAST5 files containing raw Nanopore signal data were base called and converted to FASTQ format in real-time using Guppy v3.3.0, and BBDuk v38.84 was used to trim sequences shorter with mean quality scores of less than 7 to facilitate assembly barcode and adapter sequences.

### 2.4. Genome Assembly and Annotation

The genome was assembled with Unicylcer v0.4.8. [18] providing trimmed Illumina reads as paired short reads and trimmed MinION reads as long reads. SeqSero [19] and MLST 2.0 (Multi-Locus sequence typing) [20], PlasmidFinder 2.1 [21] were used to determine *Salmonella* serotype, sequence type, and plasmid type, respectively. ResFinder 4.1 was used to determine the presence of acquired antimicrobial resistance genes and chromosomal mutations in the *gyrA*, *gyrB*, *parC*, and *parE* genes with settings of threshold of 90%, and minimum length of 60% with raw sequencing reads [22]. Pseudofinder v0.10 (https://github.com/filip-husnik/pseudofinder) (accessed on 10 March 2021), which automatically detects pseudogene candidates in prokaryotic genomes was used predict the content of pseudogenes. The assembled genome was annotated at the PATRIC annotation server with the RASTtk algorithm [23] default parameters.

SPIs were detected using BLAST against the reference sequences in previous studies [24,25] and VFDB (http://www.mgc.ac.cn/cgi-bin/VFs/search.cgi, accessed on 10 March 2021) with the threshold 50% of Grade using Geneious Prime 2020. Genomic islands (GI) were predicted by SIGIHMM, IslandPick, and IslandPath-DIMOB at islandviewer4 (https://www.pathogenomics.sfu.ca/islandviewer, accessed on 10 March 2021).

### 2.5. Phylogenetic Analysis

A total of 30 genomes of *Salmonella* spp. including the complete genome of *S. houtenae* str. 20-369, 23 complete genomes of *Salmonella* spp. (*S. bongori* str. N268, *S. enterica* subsp. *arizonae* str. CP000880, *S. enterica* subsp. *diarizonae* str. 16SA00356, 16 serotypes of *S. enterica* subsp. *enterica*, and three serotypes of *S. houtenae*), and seven draft genomes of *S. houtenae* 45:g,z51:- were used for phylogenetic analysis. The seven *S. houtenae* 45:g,z51:- strains were selected based on confirmation by Seqsero and available information on source, collection year, location, Bioproject ID and Biosample ID and their FASTA-formatted contigs were downloaded from EnteroBase (http://enterobase.warwick.ac.uk/species/index/senterica, accessed on 15 March 2021). The information on ten *S. houtenae* strains used for phylogenetic analysis is shown in Supplementary Table S1.

High-quality SNPs were identified using CSI phylogeny 1.4 and with the complete genome of *S. houtenae* str. 20-369 as a reference genome using default quality filters [26]. Maximum likelihood (ML) phylogenetic tree was constructed using RAxML-HPC v.8 with 1000 bootstrap replicates on XSEDE [27].

### 2.6. Comparative Genome Analysis

The Blast Ring Image Generator (BRIG) (v0.95) program was used to determine the genome comparison between the complete genome of *S. houtenae* str. 20-369 and the complete genomes of *S. bongori* str. N268, *S. enterica* subsp. *arizonae* str. CP000880, *S. enterica* subsp. *diarizonae* str. 16SA00356, *S. enterica* subsp. *enterica* Enteritidis (*S.* Enteritidis) str. P125109, *Salmonella enterica* subsp. *enterica* Typhimurium (*S.* Typhimurium) str. LT2, and *S. houtenae* CFSAN000552, 2009K170, and CVM 24399 strains (Table S1) from NCBI Reference sequence database [28]. The circular comparative genomic map was constructed by BRIG with standard default parameters and NCBI local blast-2.9.0+ suite.

### 2.7. Virulence Gene Profiles

Protein annotations associated with virulence were downloaded from the PATRIC workspace. The virulence profile of *S. houtenae* str. 20-369 was compared with ten *S. houtenae* strains using the virulence factor database (VFDB), a reference database for bacterial virulence factors (http://www.mgc.ac.cn/cgi-bin/VFs/genus.cgi?Genus=Salmonella, accessed on 15 March 2021) (Table S1).

## 3. Results and Discussion

### 3.1. Genomic Features

The genome of *S. houtenae* str. 20-369 has 4,651,052 bp with a G + C content of 51.7%. Annotation of the genome sequences revealed a total of 4575 putative protein-coding sequences (CDSs), 84 tRNAs, and 22 rRNAs. The isolate does not harbor any plasmids. The sequence type was ST107 by multi-locus sequence typing (MLST) analysis which is the most frequent sequence type in *S. houtenae* 45:g,z51:- in the Enterobase.

**Figure 1:**
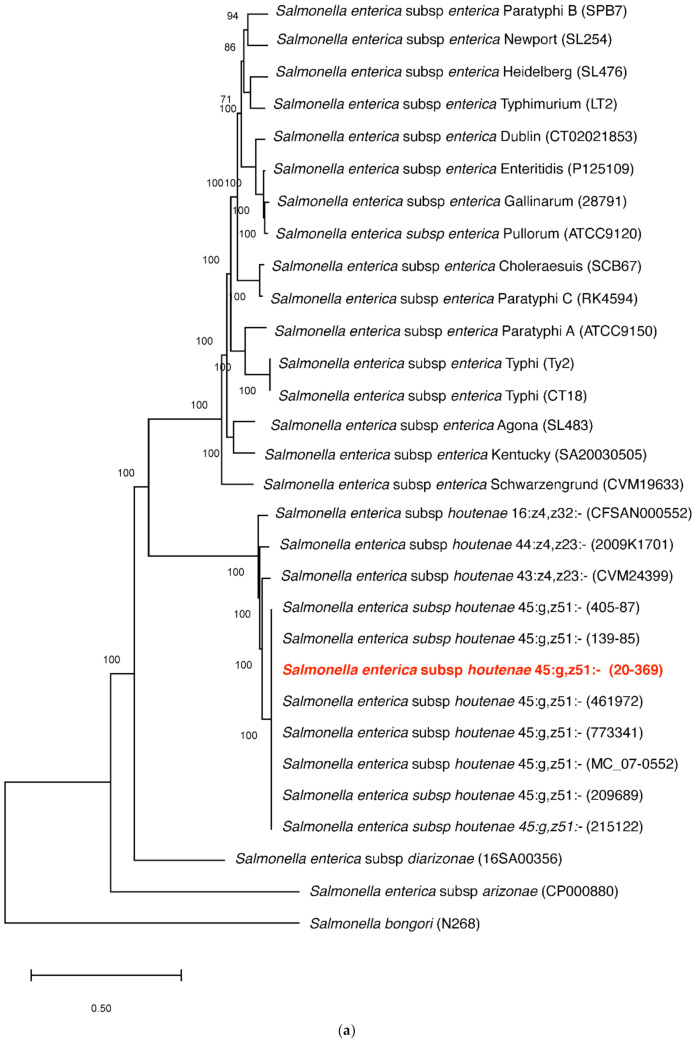

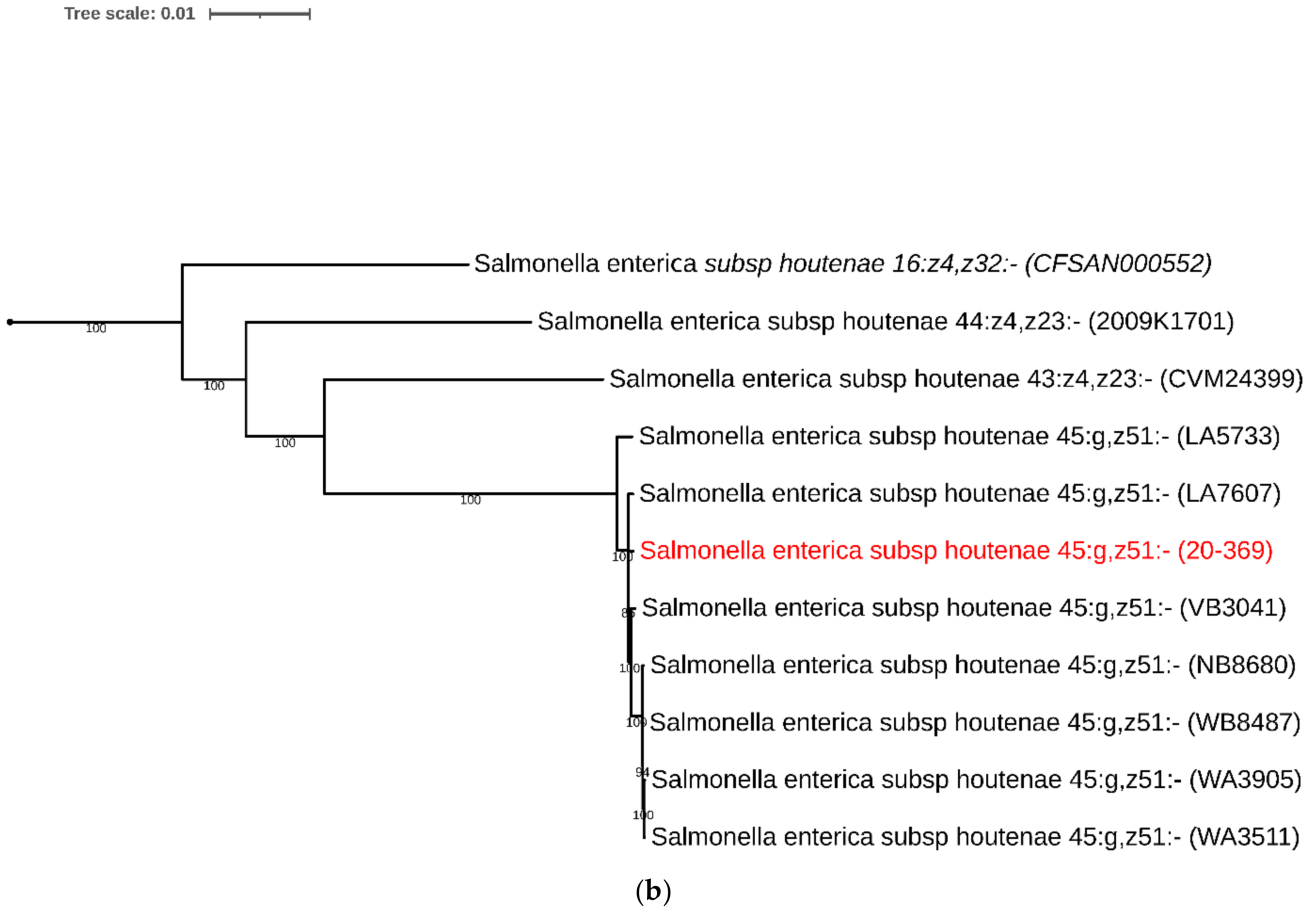
Phylogenetic analysis of 30 complete genomes of *Salmonella* spp., including strain 20-369, using SNP analysis. (**a**) The phylogeny was rooted at midpoint. (**b**) The phylogeny of *Salmonella enterica* subsp. *houtenae* strains. The scale bars show the number of substitutions per site. The numerical values represent 1000 bootstrap replicate values above 0.9.

The *S. houtenae* str. 20-369 was assigned to serotype IV 45:g,z51:- or IIIa 45:g,z51:- using the SeqSero tool (*Salmonella* Serotyping by Whole Genome Sequencing). In the maximum likelihood (ML) phylogeny, the *S. houtenae* str. 20-369 clustered with other *S. houtenae* strains, and the *S. houtenae* strains formed a well-supported monophyletic clade with high bootstrap value (Figure 1a). In a subtree (Figure 1b), the *S. houtenae* str. 20-369 clustered with the seven *S. houtenae* 45:g,z51:- strains with >97% sequence identity.

The percentage of detected pseudogene candidates was 5.75% (244 of 4241). For *Salmonella,* pseudogene accumulation has been regarded as a signature of host-specific pathogenic bacteria (*S*. Dublin with 289 pseudogenes) as compared to their host-generalist relatives (*S.* Enteritidis with 111 pseudogenes) [29]. When comparing the percentage of pseudogenes normalized to the total ORFs, our isolate contains 5.7% of pseudogenes. Similarly, *S*. Dublin which is bovine-adapted have a pseudogene content of about 5.7%, and other narrow-host-range *Salmonella* serovars like *S*. Choleraesuis (pigs), *S*. Typhi (humans), *S*. Gallinarum, and Pullorum (birds) showed a higher percentage of Pseudogenes (6.5–7.6%) in other study [30]. Therefore, available data and our results further support host-adaptation of *S. houtenae* to reptile.

### 3.2. Antibiotic Resistance

*S. houtenae* str. 20-369 was susceptible to all antibiotics tested except streptomycin (aminoglycosides) and carries the *aac(6′)-Iaa* gene which is a chromosomal-encoded aminoglycoside 6′ N-acetyltransferase and a point mutation T57S in the *parC* gene, but mutations were not observed in *gyrA*, *gyrB*, or *parE* genes.

In relation to the antimicrobial susceptibility of *Salmonella*, there are few data concerning the antimicrobial resistance of *S. houtenae* compared with *S. enterica* subspecies *enterica.* It has been observed that *S. houtenae* strain isolated from reptiles have resistance to antimicrobials, two *S. houtenae* isolates from gecko resistant to ampicillin or tetracycline [31] and *S. houtenae* 45:g,z51:- isolated from healthy captive bred female veiled chameleons resistant to streptomycin [32]. In the previous study of the whole genome analysis of *S. houtenae* [14], the isolate shows the susceptible phenotype to all antimicrobials tested but carries the antimicrobial resistance gene associated with aminoglycoside resistance (*Aac6-Iaa_AGly*), which it could be under-expressed. In our study, phenotypic antimicrobial resistance was concordant with genotypic antimicrobial resistance for streptomycin but not for quinolone. It has been reported that the mutations in the *parC* are not essential for quinolone resistance, but it can result in a high level of fluoroquinolone resistance [33].

### 3.3. Genomic Islands (GI) and Salmonella Pathogenicity Islands (SPI)

**Figure 2:**
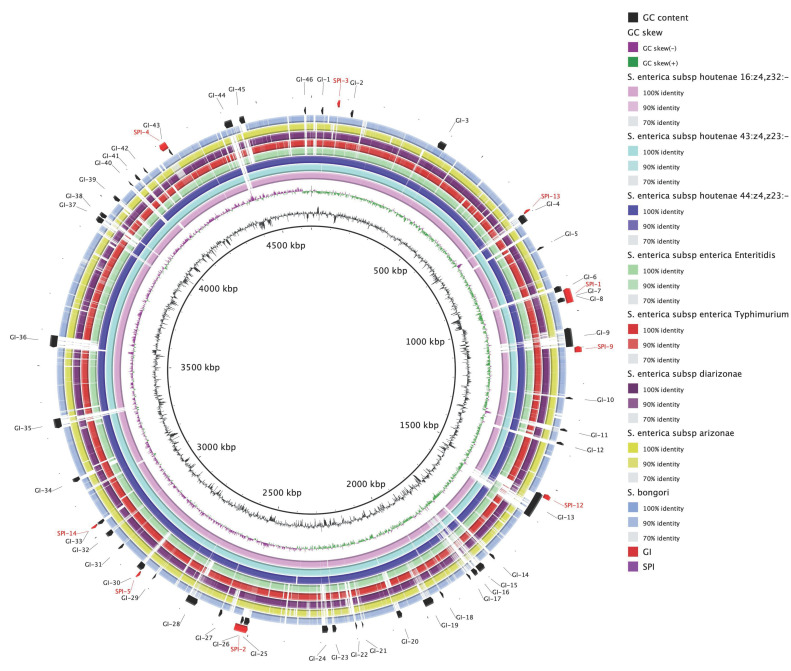
Blast Ring Image Generator (BRIG) diagram showing the complete genomes of *Salmonella* spp. strains, with genome of strain 20-369 as a reference. The outer circle contains genomic island regions (black) and *Salmonella* Pathogenicity Island (SPI) (red) of strain 20-369. GC content is also shown in the figure.

A total of 46 GIs, designated as GI-1 to GI-46, were predicted using SIGIHMM, IslandPick and IslandPath-DIMOB (Figure 2, Table S2). By comparing the sequence-based similarity of the genomes using an all-against-all BLAST comparison, we identified six novel islands (GI-19, GI-21, GI-22, GI-24, GI-36, and GI-46) observed only in the complete genomes of *S. houtenae* strains (Figure 2). Detailed inspection of these islands revealed the presence of predicted proteins related to antitoxin HigA, toxin HigB, fimbrial proteins, transposase, phage-related protein, error-prone repair protein, oxidoreductase, transcriptional regulator (Lys family, GntR family), Type VI secretion system (T6SS), and phosphotransferase (PTS) system (Table S2). In addition, specific portions of seven novel genomic islands (GI-6, GI-14, GI-15, GI-16, GI-18, GI-30, and GI-32) were found in *S. houtenae* str. 20-369 but absent in the rest of the genomes analyzed (Figure 2). These novel genomic islands contain the genes encoding the proteins associated with type III secretion system (TTSS), antitoxin HigA, toxin HigB, transposase, sugar transferase, error-prone repair protein, isochorismatase family protein, and hypothetical proteins (Table S2).

In a previous study [14], *S. houtenae* str. CFSAN039533 shared 234 (86%) of selected proteins with *S.* Enteritidis str. 77-1427 and *S.* Typhimurium str. LT2, but three genes related to virulence functions (*ClfA*) and regulation systems (*HigA, Ygfl*) were classified as specific for CFSAN039533 strain. In our study, the genomic islands contain the genes encoding the HigB/HigA toxin/antitoxin system which is one of the toxin-antitoxin (TA) systems, which are prevalent in most bacterial and archaeal genomes, and one of the emerging physiological roles of TA systems is to help regulate pathogenicity [34]. Genes for the HigB/HigA TA systemare found in the chromosomes of the many pathogens such as *Vibrio cholera*, *Streptococcus pneumoniae*, *Acinetobacter baumannii*, *Yersinia pestis*, *E. coli* CFT073 and *E. coli* O157:H7 [34], but not reported in *Salmonella* spp. [35,36].

BLAST results showed that all three isolates harbor major SPIs, including SPI-1 to SPI-5, SPI-9, SPI-12 to SPI-14 (Figure 2). The SPI-1 to SPI-5 has been described as main SPIs of *Salmonella* genome [37], and many studies have reported that SPI-1, SPI-2 and SPI-4 are conserved genetic islands, while SPI-3 and SPI-5 displayed a variable genetic information [14]. In the previous study [14], the CFSAN039533 strain displayed virulence genes belonging to SPI-1, SPI-2, SPI-3 and SPI-5 when they performed a virulence gene database constituted with representative genes of SPIs (SPI-1 to SPI-5 and SPI-7), but the strain did not carry *siiD* and *siiE* of SPI-4. However, in this study all *S. houtenae* strains carry *siiD* but none of them carries *siiF* (data not shown). All *S. houtenae* 45:g,z51:- strains except the 20-369 and MC_07-0552 strains carry *siiE* (data not shown).

### 3.4. Virulence Gene Profile

**Figure 3:**
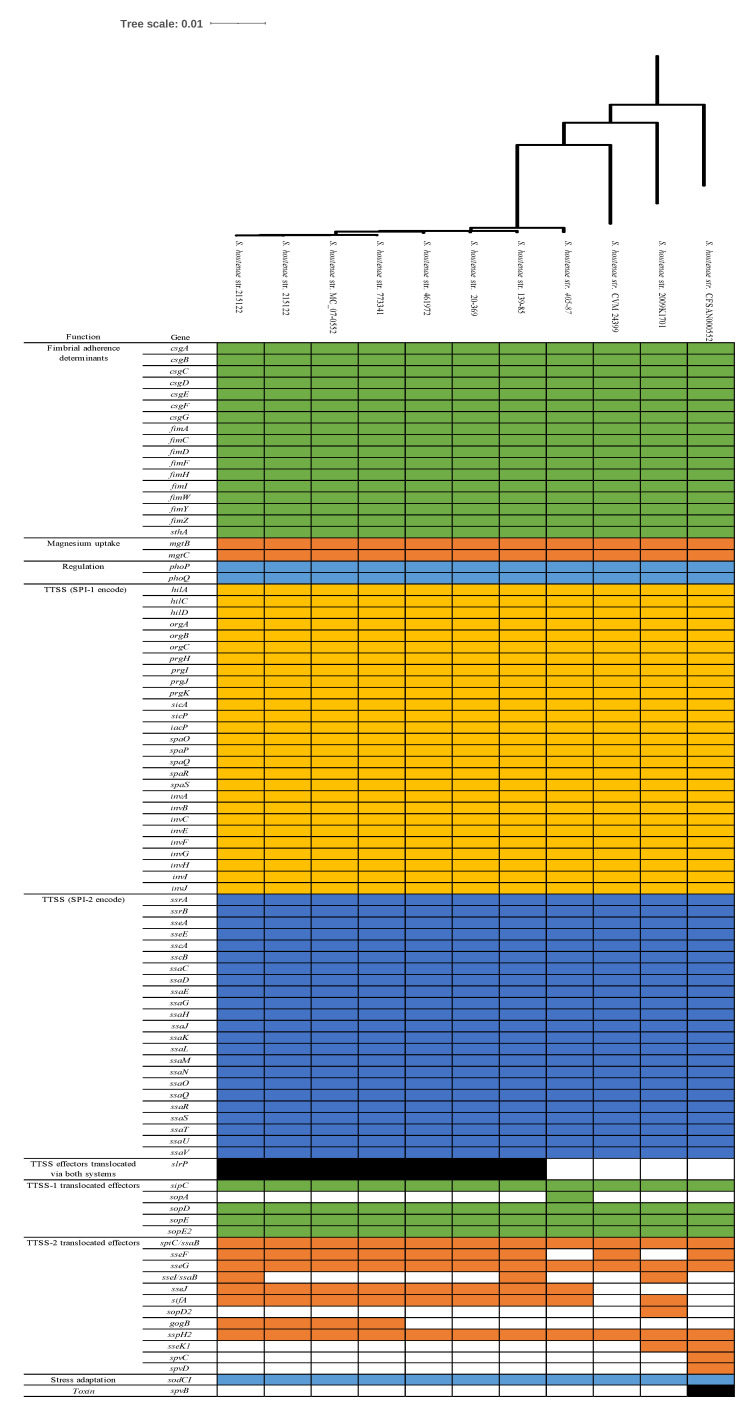
Virulence gene profile of 11 *Salmonella enterica* subsp. *houtenae* strains including strain 20-369.

Genome sequences of 11 *S. houtenae* strains including our isolate were analyzed for virulence genes using the VFDB (Figure 3). The *S. houtenae* strains carry a conserved virulence gene profile in fimbrial adherence determinants (*csg, fim*, and *sthA*), magnesium uptake genes (*mgtB* and *mgtC*), regulation (*phoP* and *phoQ*), TTSS (SPI-1 and SPI-2 encode), and stress adaptation (*sodCl*) but show various profiles in TTSS effectors translocated via both systems (*slrp*), TTSS-1 translocated effectors (*sopA*), TTSS-2 translocated effectors (*sseF*, *sseI/ssaB*, *sseJ*, *sifA*, *sopD2*, *gog*, *B sseK1*, *spvC*, and *spvD*) and toxin (*spvB*). Only the *S. houtenae* 16:z4,z32:- str. CFSAN000552 carries *spvB*, *spvC*, and *spvD*.

Eight *S. houtenae* 45:g,z51:- showed identical virulence gene profile except in five virulence genes; *slrp,* *sopA*, *sseF, sseI/ssaB* and *gogB*. Compared to other *S. houtenae* isolates, *S. houtenae* str. 20-369 does not carry seven virulence genes associated TTSS translocated effectors (*sopA*, *sseI/ssaB, sopD2*, gogB, *sseK1*, *spvC*, and *spvD*) and a toxin gene *spvB* (Figure 3). The *S. houtenae* str. 20-369 has an identical virulence gene profile with *S. houtenae* 45:g,z51:- str. 461972, which was isolated from human in United Kingdom in 2006 (Figure 3 and Table S1), of which supports that *S. houtenae* is an opportunistic pathogen.

It is notable that the cases found for *S. houtenae* infections have been focused on the brain, and it appears that this subspecies has an affinity for the brain, causing extra-intestinal infections [1]. However, due to a lack of available complete genome sequences of *S. houtenae*, the genome comparison study for genetic relatedness and pathogenic mechanism could not be performed in this study.

In this study, we announced the first complete and closed genome sequence of *S. houtenae* 45:g,z51:- isolated from a reptile in United States and analyzed the antibiotic resistance and virulence gene profiles. Overall, this study found a number of interesting genomic features such as pseudogene formation and novel genomic islands. With this study, we provide an importance basis for an understanding of the genetic mechanism that underlies pathogenicity *S. houtenae* 45:g,z51:-, as well as a high-quality reference for future genome comparison studies.

## Figures and Tables

**Table 1:** Antibiotic resistance phenotype and genotype of *S. houtenae* str.20-369 isolate of this study.

Type	Antibiotics	Antibiotic Resistance *
Genotype	Aminoglycoside	aac(6′)-laa,
	Fluroquinolone	-
Phenotype	Gentamicin (Aminoglycosides)	S
	Streptomycin (Aminoglycosides)	R
	Amoxicillin clavulanate (B-lactam combination)	S
	Cefoxitin (Cephems)	S
	Ceftiofur (Cephems)	S
	Ceftriaxone (Cephems)	S
	Ampicillin (Penicillin)	S
	Imipenem (Carbapenems)	S
	Sulfisoxazole (Folate pathway antagonist)	S
	Trimethoprim-sulfamethoxazole (Folate pathway antagonist)	S
	Chloramphenicol (Phenicols)	S
	Ciprofloxacin (Quinolones)	S
	Enterofloxacin (Quinolones)	S
	Marbofloxacin (Quinolones)	S
	Nalidixic acid (Quinolones)	S
	Tetracycline (Tetracyclines)	S
	Nitrofurantoin (Nitrofurans)	S

* S, sensitive; R, resistance.

## Data Availability

The complete genome sequence of the isolate 20-369 in this study have been deposited in the National Center for Biotechnology Information (NCBI)’s under the Bioproject accession number PRJNA725952.

## Supplementary Materials

The following are available online at https://www.mdpi.com/article/10.3390/antibiotics10111322/s1, Table S1. List of *Salmonella enterica* subsp. *houtenae* strains downloaded as FASTA-formatted contigs from NCBI and EnteroBase analyzed in this study (*n* = 10), Table S2. The genomic islands (GI) and annotation of the strain 20-369.
